# Peripheral blood gene expression signatures of systemic immunity predict tumor microenvironment biology and therapeutic response in breast cancer

**DOI:** 10.21203/rs.3.rs-6926989/v1

**Published:** 2025-07-07

**Authors:** Justin Balko, Xiaopeng Sun, Ann Hanna, Andres Ocampo, Brandie Taylor, Jacey Marshall, Julia Steele, Margaret Axelrod, Elizabeth Wescott, Susan Opalenik, Angela DeMichele, Laura Esserman, Minetta Liu, Rita Nanda, Denise Wolf, Lamorna Brown Swigart, Gillian Hirst, Laura Van ‘T Veer, Yaomin Xu

**Affiliations:** Vanderbilt University Medical Center; Vanderbilt University Medical Center; Vanderbilt University Medical Center; Vanderbilt University Medical Center; Vanderbilt University Medical Center; Vanderbilt University Medical Center; Vanderbilt University Medical Center; Washington University in St Louis; Vanderbilt University Medical Center; Vanderbilt University Medical Center; University of Pennsylvania; University of California, San Francisco; Natera Inc.; University of Chicago; University of California, San Francisco; University of California San Francisco; University of California, San Francisco; UCSF; Vanderbilt University Medical Center

**Keywords:** Breast cancer, immune checkpoint inhibitor, peripheral blood, biomarker

## Abstract

This study investigates the association between peripheral blood immunological features and immunotherapy response in breast cancer. We generated and analyzed RNAseq data from 546 blood samples of patients with high-risk stage II/III HER2-negative breast cancer enrolled in the I-SPY2 trial and identified peripheral immune signatures associated with tumor characteristics and immunotherapy response. Triple negative breast cancer (TNBC) patients showed higher T cell receptor (TCR) clonality and immune activation signatures. Responders to the chemotherapy + pembrolizumab regimen had high baseline TCR diversity, with TNBC responders experiencing T cell clonal expansion and activation after treatment. A logistic regression model based on immunological features before and early-on-treatment predicted response to pembrolizumab. The model was validated in an independent cohort of patients treated with dostarlimab in the neoadjuvant setting. We report the potential of peripheral blood-derived gene expression tests to predict immunotherapy benefit, guiding personalized treatment in breast cancer with a minimally invasive approach.

## Introduction

Breast cancer remains one of the leading causes of cancer and cancer-related death among females in the United States. For localized disease, breast cancer subtype guides the standard systemic therapy administered. Hormone receptor positive (HR + BC) patients are commonly treated with endocrine therapy, with a fraction of patients benefiting from additional chemotherapy treatment. Patients bearing HER2 + tumors are often treated with trastuzumab-based targeted therapy. Historically, treatment options for triple-negative breast cancer (TNBC) have been limited to chemotherapy and radiation therapy due to the absence of HR and HER2.

Recently, the landscape of TNBC treatment has undergone a paradigm shift with the emergence of immunotherapy. Immunotherapy, particularly immune checkpoint inhibitors (ICI), has become a standard of care in the management of early-stage TNBC, improving pathological complete response (pCR) rates and event-free survival^1–3^. Interest in combining ICI with chemotherapy in HR+/HER2− breast cancer has also received significant attention as recent studies demonstrated significant increase in pCR rate in early high-risk HR+/HER2− breast cancer^2,4^. Despite the promising outcomes observed in several phase III clinical trials, the addition of immunotherapy only benefits a limited fraction of patients, exposing a large fraction of the patients to potentially life-long and life-threatening immune-related toxicities and undue financial burden with limited benefit. Thus, there exists a pressing need to improve upon the current biomarkers to optimize therapy in patients. Tissue based biomarkers, like tumor-specific MHC-II expression, have been validated retrospectively in a large number of studies across multiple groups^5–10^. However, other biomarkers such as PD-L1 immunohistochemistry and tumor-infiltrating lymphocytes have proven to be generally prognostic or to have limited value in predicting immunotherapy-specific outcomes^1,3,11,12^.

While tumor biopsy tissue remains a valuable source for biomarker identification, its utility is challenged by its invasive nature and difficulties in obtaining longitudinal samples. Longitudinal sampling (e.g. before and early after therapy initiation) can provide substantially more information on patient-specific therapeutic response and reduce interpatient variability. As anti-tumor immunity evolves dynamically during tumor progression and exposure to therapy, the utility of a liquid biopsy presents a compelling alternative, offering a minimally invasive tissue source amenable to longitudinal sampling and evaluation of individualized response to therapy.

Peripheral immunological biomarkers have demonstrated utility in predicting immunotherapy response across diverse cancer types. In melanoma and non-small cell lung cancer, abundance of specific immune cell subsets and neutrophil/monocyte to lymphocyte ratios correlate with patient response and prognosis^13–16^. However, the development of such biomarkers could be improved by enhancing our understanding of the behavior of the systemic anti-tumor immune response during therapy.

To address these critical gaps, we performed bulk RNA sequencing on a discovery set of 549 longitudinal buffy-coat blood samples obtained from 160 patients enrolled in the I-SPY2 paclitaxel control arm and ‘pembro4’ (pembrolizumab + paclitaxel) treatment arm (followed by standard doxorubicin and cyclophosphamide in both arms). Our analysis focused on identifying peripheral transcriptomic profiles and immune cell compositions associated with breast cancer subtype, treatment regimen, and treatment response. Additionally, we investigated the correlation between peripheral immunity and the tumor immune microenvironment using matched baseline tumor biopsies from the original report^17^. Our findings suggest that a composite assessment of peripheral blood gene expression may hold promise as a dynamic biomarker for deployment in future immunotherapy trials, and the potential for a novel type of liquid biopsy that informs upon the systemic response to cancer therapy.

## Results

### Patients and samples

**Figure 1:**
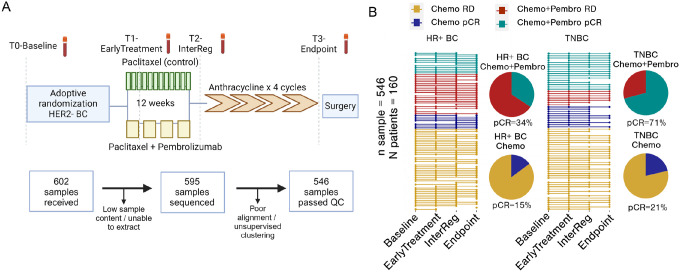
I-SPY2 peripheral blood collection timepoint and sample distribution. **(A)** Longitudinal buffy coat blood samples from patients enrolled in the I-SPY2 Chemo control arm and Chemo+Pembro treatment arm were collected at baseline, after 1 cycle of therapy (EarlyTreatment), after tested regimen and before anthracycline (InterReg) and endpoint. **(B)** In total, 546 RNA samples from 160 patients passed quality control. Samples involved in the subsequent analysis are depicted here with patients’ pCR rate separated by receptor type and treatment arm.

We processed 546 longitudinal buffy coat blood samples from 160 patients with high-risk stage II/III HER2-negative breast cancer enrolled in the I-SPY2 clinical trial [**Supplementary Table 1**]. Buffy coat blood samples were obtained at baseline (T0), after one cycle of therapy (T1; Early Treatment), between the study treatment and the standard universal treatment of doxorubicin and cyclophosphamide (T2; InterReg), and therapy endpoint (T3; Endpoint/Surgery) [Figure 1A]. There were 89 HR + BC patients and 71 TNBC patients. Each group was further subdivided into those receiving neoadjuvant paclitaxel (control; Chemo) and those receiving neoadjuvant paclitaxel + pembrolizumab (Chemo + Pembro). Treatment details and patient selection criteria have previously been reported^18^.

Patient responses were classified as either pCR or residual disease (RD) based on the residual cancer burden. Among the HR + BC patients, 54 cases were treated with Chemo, with 8 achieving pCR and 46 having RD (14.8% pCR rate vs. 13% reported previously). 35 HR + breast cancer received Chemo + Pembro, with 12 achieving pCR and 23 having RD (34.2% pCR rate vs. 30% reported previously). For the TNBC group, 47 patients were treated with Chemo, with 10 achieving pCR and 37 having RD (21.2% pCR rate vs. 22% reported previously). For TNBC patients treated with Chemo + Pembro, there were 24 patients, with 17 achieving pCR and 7 having RD (70.8% pCR rate vs. 60% reported previously) [Figure 1B]. Compared to the previously published clinical results from the I-SPY2 study^2^, our analyzed cohort shows a slight enrichment for pembrolizumab responders.

In the biomarker validation cohort, we performed RNAseq on 139 longitudinal buffy coat blood samples from 77 HR + or TNBC patients receiving oral paclitaxel + encequidar + carboplatin + dostarlimab (Dostar Arm). The treatment details and patient selection criteria has been reported^19^. There were 59 patients (20 HR + BC [7 pCR patients], 39 TNBC [23 pCR patients]) with paired Baseline and EarlyTreatment samples

### Baseline peripheral immune signatures differ by breast cancer subtypes

**Figure 2:**
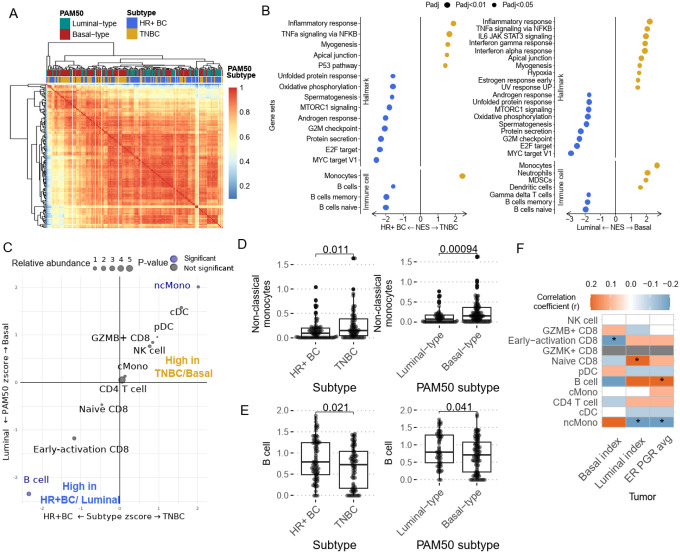
Baseline peripheral immune profile is different between breast cancer subtype. **(A)** Correlation analysis was performed between each individual samples using the top 5000 most differentially expressed genes. Then, unsupervised clustering based on the correlation matrix was performed. The heatmap depicts the correlation coefficient. **(B)** Significantly enriched hallmark gene sets and peripheral immune cell phenotype gene sets in HR+BC/Luminal-like (blue) and TNBC/Basal-like (gold). Only pathways with GSEA permutation test adjusted p value < 0.05 are presented here. BH p-value adjustment was performed. **(C)**Association between CIBERSORT derived immune cell abundance and PAM50 / clinical subtype calculated with logistic regression. A positive logistic regression z score for PAM50(y axis) or clinical subtype (x axis) indicates the cell type is more abundant in Basal-like tumor or TNBC tumor, respectively. Negative z score indicate the cell type is more abundant in Luminal-like or HR+ BC. CIBERSORT deconvoluted **(D)** non-classical monocyte and (E) B cell abundance were compared between breast cancer clinical(left panels) and PAM50 subtypes (right panels) witht-test. Numerical p-value is reported here **(F)**The association of immune cell abundance and lineage phenotype and tumoral HR expression demonstrated by Pearson correlation. Correlation with numerical p value<0.05 are highlighted with Asterisk.

To determine how the immune signatures differ by breast cancer subtype, the peripheral immune landscape was first analyzed at baseline. Using unsupervised clustering on the top 5000 most variable genes, we observed a trend where peripheral blood transcriptomic signatures were clustered according to the tumor’s PAM50 subtype, and to a lesser extent, by clinical subtype [Figure 2A]. We compared the average correlation coefficient within blood from luminal-like breast cancer and between luminal and basal-like breast cancer. There is a higher average correlation coefficient within the subtype than across subtypes [**Supplementary Fig. 1A**]. It was noted that the clustering trends from the peripheral blood were less pronounced than the trends observed when analyzing RNA derived directly from the tumor^20^. Thus, peripheral immune characteristics are associated with specific breast cancer subtypes, though these differences are more subtle than those observed directly from the tumor, as expected. Nonetheless, it was surprising and intriguing that even nuanced gene expression profiles could be observed peripherally based on underlying breast cancer biology.

To further investigate whether the breast cancer clinical and molecular subtype is associated with systemic immune response, we performed differential gene expression analysis between baseline samples from TNBC and HR + breast cancer patients, followed by gene set enrichment analysis (GSEA) with HALLMARK pathways. We observed a significant enrichment (GSEA permutation test adjusted p value < 0.05) of “inflammatory response” and “TNFa signaling” in TNBC patients compared to HR + BC [Figure 2B]. Similar upregulation of immune response signals was observed when comparing between molecular subtypes, with higher inflammatory and interferon gamma response signals observed in Basal-like breast cancer patients. As TNBC/Basal-like tumors are known to be more immunogenic^21,22^, this observation was expected, however was novel to observe in peripheral blood cells. To better understand the underlying immune cell composition differences, we quantified the peripheral immune cell by performing GSEA using immune cell reference genes from the PanglaoDB single-cell database. We observed a significant enrichment (GSEA permutation test adjusted p value < 0.05) of myeloid cell genes, such as those associated with monocytes, neutrophils and myeloid derived suppressor cells (MDSCs) in TNBC/Basal-like breast cancer patients, whereas B cell-associated genes were upregulated in HR + BC/Luminal breast cancer patients [Figure 2B]. In addition, we applied CIBERSORTx immune cell deconvolution algorithm using our previously published breast cancer PBMC single-cell RNA sequencing data [**Manuscript under review**] as reference to further validate the differences in PBMC composition. We were able to corroborate the findings from GSEA, with B cells significantly more abundant in the PBMC from HR+/ luminal-like BC, while non-classical monocytes are significantly higher in TN/Basal-like BC [Figure 2 C–E]. Since both breast cancer molecular subtype and clinical subtype are derived from a continuous scale of basal/luminal transcriptomic signatures or HR expression, respectively, we also calculated the correlation between PBMC immune cell abundance, basal/luminal status, and ER/PR expression level that is previously documented by the I-SPY2 tumor analysis^17^. Besides B cells and monocytes, we also observed a significant positive correlation between luminal score and the abundance of naïve CD8 T cells, which is negatively associated with basal lineage score. Interestingly, the GZMB + cytotoxic CD8 cell abundance is positively associated with basal score [Figure 2F]. Profiling the T cell receptor (TCR) repertoire has proven to be an important method to measure autologous or treatment-induced antitumor immune response^23,24^. We compared TCR clonality between breast cancer subtypes to assess their baseline adaptive immune response. When comparing the TCR Shannon diversity between the two subtypes, we observed a lower Shannon diversity index in TNBC/Basal-like BC patients, suggesting a greater abundance of clonal T cells in their PBMCs [**Supplementary Fig. 2 A, B**]. These results suggest that patients with TNBC/Basal-like tumors tend to have a more inflammatory systemic immune response, potentially driven by monocytes and clonal expansion of T cells.

To further validate peripheral immune signature differences between breast cancer subtypes, we performed resampling (bootstrapping) 50 times on all available baseline samples (128 HR + BC and 132 TNBC; samples from Pembro4, Dostar, and paclitaxel control arms) to generate various breast cancer subsamples. Differential gene expression analysis and subsequent gene set enrichment analysis were performed to detect peripheral immune signatures associated with breast cancer subtypes in each iteration. This method allows us to measure the stability and variability of the differential gene analysis and reduce sample-specific bias. If a pathway is consistently enriched/de-enriched during multiple subsampling, they are more likely to be truly different between the breast cancer subtypes. Although the enrichment of Hallmark pathway signatures was found to be relatively noisy during the resampling process, we found very consistent enrichment of B cell signatures in HR + BC and T cell signatures in TNBC [**Supplementary Fig. 1B**]. This data, along with the differential gene expression analysis, suggest that baseline peripheral blood transcriptomic signatures truly differ by breast cancer subtype.

### Comparing the peripheral and tumor immune landscapes

Previous I-SPY2 studies sought to characterize pre-treatment tumor signaling and immune landscape features using a series of mechanism of action biomarkers^17^. In that study, 8 immune phenotype scores were generated to describe the tumor immune microenvironment. All these signatures, except mast cell score, were higher in pCR than RD cases in most of the treatment arms. In the HR + HER2 − subtype, the mast cell signature was higher in RD cases, mainly due to its negative association with pCR in the pembrolizumab arm. To further investigate the association between tumor and peripheral immune response, we performed a correlation analysis between blood-based immune signatures and the eight continuous tumor-based immune biomarkers derived from patient-matched baseline tumor biopsy and peripheral blood samples.

**Figure 3:**
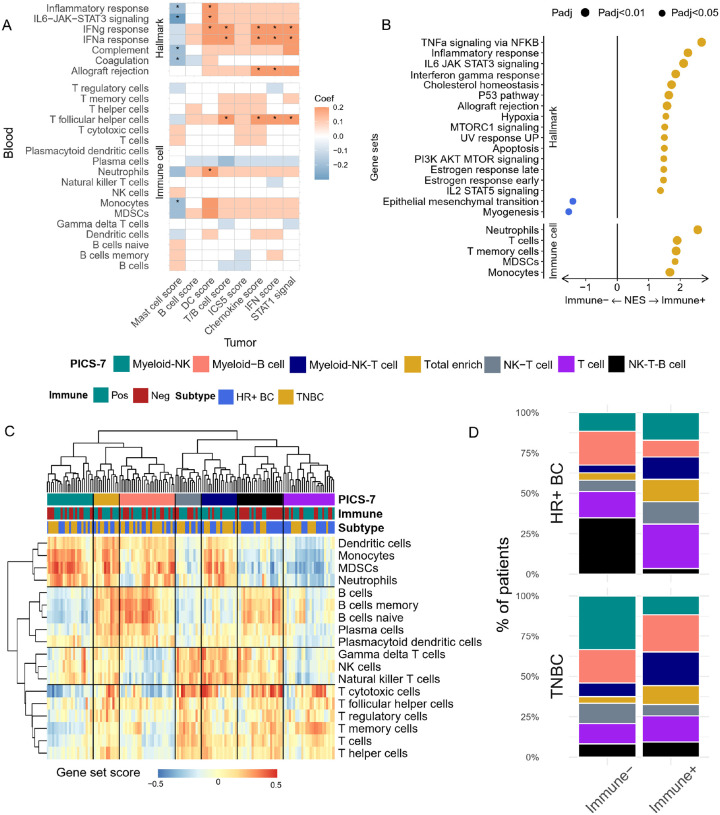
Tumor immune profiles are associated with peripheral immune signatures. **(A)** Correlation analysis was performed between the peripheral blood immune signatures measured by GSVA of Hallmark pathways and immune cell subtype, and tumor immune composite score derived from patient’s tumor microarray data. Correlations with numerical p-value<0.05 were labeled with Asterisk. **(B)** Gene set enrichment analysis was performed using the differentially expressed genes in Immune+ and Immune− tumors. Significantly enriched hallmark pathways and peripheral immune cell phenotype gene sets in Immune+ (right, gold) and Immune− (left, blue) patient’s blood, pathways with GSEA permutation test adjusted p value < 0.05 are presented here. BH p-value adjustment was performed. **(C)** GSVA was performed on each Baseline and EarlyTreatment samples to obtain the enrichment score for different immune cell subtypes. The immune cell scores were depicted in the heatmap. Hierarchical clustering on the immune cell scores reveals 7 distinct PBMC immune composition subtype (PICS7). **(D)** The patients’ baseline blood sample were classified into PICS-7. The abundance of each PICS-7 subtype in the Immune+ and Immune− groups were measured separately by breast cancer clinical subtype.

Peripheral immune signatures were quantified using 1) immune related pathways from “Hallmark” pathways and 2) immune cell signatures from PanglaoDB. Our analysis revealed that interferon response in the blood was positively associated with positive response predictors such as tumor chemokine signaling, T and B cell scores, STAT1 signaling, and tumor interferon scores [Figure 3A]. Conversely, a negative correlation was observed between the mast cell score and several peripheral immune signals, including monocyte abundance, inflammatory response, IL6-STAT3 signaling, and complement signal. This observation further supported the concordance in immunological signals between the tumor and periphery.

To classify a patient’s tumor immune landscape with the eventual goal of developing a tumor-based transcriptomic signature to predict therapeutic response, the previous I-SPY2 biomarker study also developed the categorical variable “Immune+” to identify immunogenic tumors^17^. In brief, Immune + definition is based on a variety of different subtype-specific signatures (e.g. B cell signature in HR+, T-B cell/STAT1/chemokine signature in TN). Patients defined as Immune + all demonstrate higher pCR rate to Chemo + Pembro regimen. We next aimed to determine if Immune + patients also exhibit a distinct peripheral immune landscape. Using differential gene expression analysis and GSEA, adjusted for clinical subtype, we found enrichment in multiple immune response pathways, including interferon, inflammatory, and interleukin responses, in Immune + patients [Figure 3B]. Additionally, these patients showed higher T cell and monocyte abundances [Figure 3B]. However, TCR clonality was not significantly different between Immune + and Immune− patients [**Supplementary Fig. 2C**]. We then performed unsupervised clustering of PBMC composition based on the score of 18 peripheral immune cell subtypes to simplify the deconvolution of the peripheral immune landscape. Seven distinct clusters were identified based on the relative enrichment score of major immune cell clusters-T cells, myeloid cells, B cells and NK cells [Figure 3C, **Supplementary Fig. 3A-E, Supplementary Table 2**]. When comparing the differences in PBMC immune composition subtypes (PICS-7) between Immune+/− patients, we found an enrichment of B cell associated clusters (Myeloid-B cell and NK-T-B cell cluster) within HR+/Immune− patients [Figure 3D]. In contrast, T cell associated clusters (T cell, NK-T cell and myeloid-NK-T cell) were enriched in HR+/Immune + patients [Figure 3D]. In TNBC, the PBMC composition difference was less pronounced, with Immune− patients predominating in the myeloid-NK clusters, while more Immune + patients were assigned Myeloid-NK-T cell clusters [Figure 3D].

### Chemo + Pembro induces systemic immune activation

Longitudinal peripheral blood samples collected in this study offer a unique opportunity to monitor the dynamics of patients’ systemic immunity while undergoing therapy. We performed a time-series analysis on the transcriptomic signatures to identify general therapeutic effects on systemic immunity induced by Chemo or Chemo + Pembro, regardless of response.

**Figure 4:**
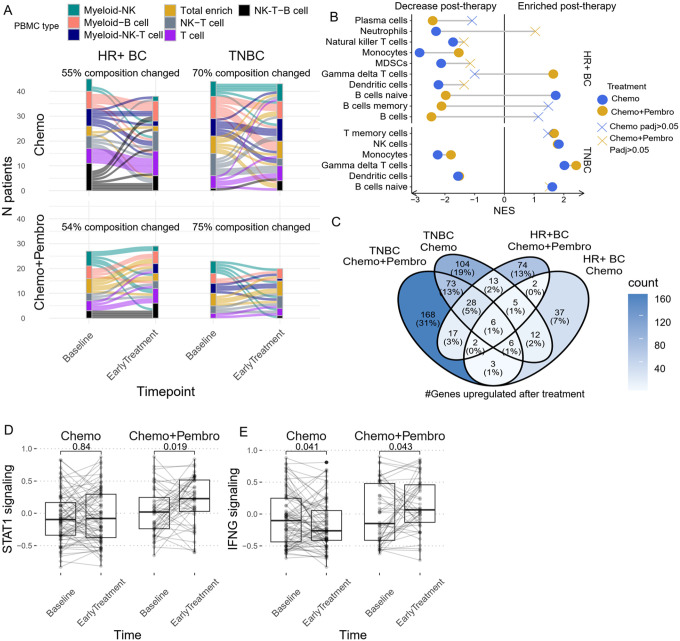
Chemo and Chemo+Pembro induced different peripheral immune signatures alteration. **(A)** PBMC composition dynamic after Chemo or Chemo+Pembro treatment summarized by PICS-7 clusters, separated by breast cancer clinical subtype. If a patient’s PBMC was classified differently before and after treatment, the patient would be classified as “composition changed”. The percentage of samples experienced composition change was measured. **(B)** Differential gene expression analysis was performed between Baseline and EarlyTreatment samples followed by gene set enrichment analysis using immune cell signature. A positive net enrichment score indicate this specific immune cell expanded after treatment. Plot is separated by clinical subtype. Immune cell signatures with adjusted pvalue>0.05 are depicted with X. **(C)** Venn diagram of genes significantly upregulated after first round of therapy grouped by treatment and breast cancer clinical subtype. Genes with numerical p value<0.01 and log2FoldChange >1 are considered significant. **(D)** STAT1 signaling, and **(E)** interferon response, two major pro-inflammatory signaling pathway was compared before and after treatment among all patients treated with chemotherapy(left) and those treated with chemotherapy + pembrolizumab (right). Paired t-test was used.

We first analyzed changes in PBMC composition using our developed PICS-7. We observed a numerically higher proportion of TNBC patients being assigned to a different PICS-7 cluster after either chemotherapy or chemotherapy + pembrolizumab. (Chemo: 70% vs 55%, Chemo + Pembro: 75% vs 54%) [Figure 4A], suggesting a greater therapy-induced systemic immune response. Interestingly, the two treatment regimens had opposite effects on B cell abundance, with Chemo + Pembro significantly reducing B cell associated signatures in HR + BC as reflected by GSEA [Figure 4B]. In TNBC, the treatment effects on immune cell composition were largely identical between the treatment arms. We observed an upregulation of B cell, NK cell, and T cell signatures post-treatment, accompanied by a decrease in myeloid cell abundance [Figure 4A, B]. One unique feature of TNBC patients treated with Chemo + Pembro was the transformation of the Myeloid-NK PICS-7 cluster post-therapy to B cell or T cell-rich clusters, which may depict a greater lymphocyte activation effect upon the addition of immunotherapy.

When comparing the overall genes between early treatment versus baseline, we observed more genes differentially upregulated in Chemo + Pembro treated TNBC patients, followed by Chemo treated TNBC patients, Chemo + Pembro treated HR + BC and Chemo treated HR + BC [Figure 4C]. There were limited overlaps among the upregulated genes within those treatment-subtype groups, hinting at the possible existence of divergent treatment-tumor interactions in the systemic immune response. We further focused the analysis on general immune activation signatures derived from the Hallmark gene set of immune signaling gene sets previously developed by the I-SPY2 biomarker study. STAT1 signaling and interferon response were significantly upregulated after Chemo + Pembro treatment [Figure 4D, E]. In contrast, the interferon response was downregulated after Chemo treatment [Figure 4E]. Both Chemo and Chemo + Pembro induced significant downregulation of inflammatory transcriptional signals. [**Supplementary Fig. 4 A, B**]. Although these differences were significant, we also found a certain level of interpatient heterogeneity in these responses to treatment, potentially contributed by the response to therapy and/or patient/disease-specific covariates.

### Peripheral cytotoxic T cell signals are associated with Chemo + Pembro response in TNBC

Although we observed unique systemic changes induced by the two treatment regimens, the overall signals were relatively inconsistent, potentially due to varied patient (or tumor subtype) response to therapy. To gain deeper insights into this variability, we next focused on how peripheral immune markers change during therapy in pCR and RD patients, specifically those treated with Chemo + Pembro. By analyzing the dynamics of the peripheral immune landscape between these two groups, we aimed to identify key markers associated with therapeutic outcomes and improve our understanding of the mechanisms underlying effective immunotherapy responses.

**Figure 5:**
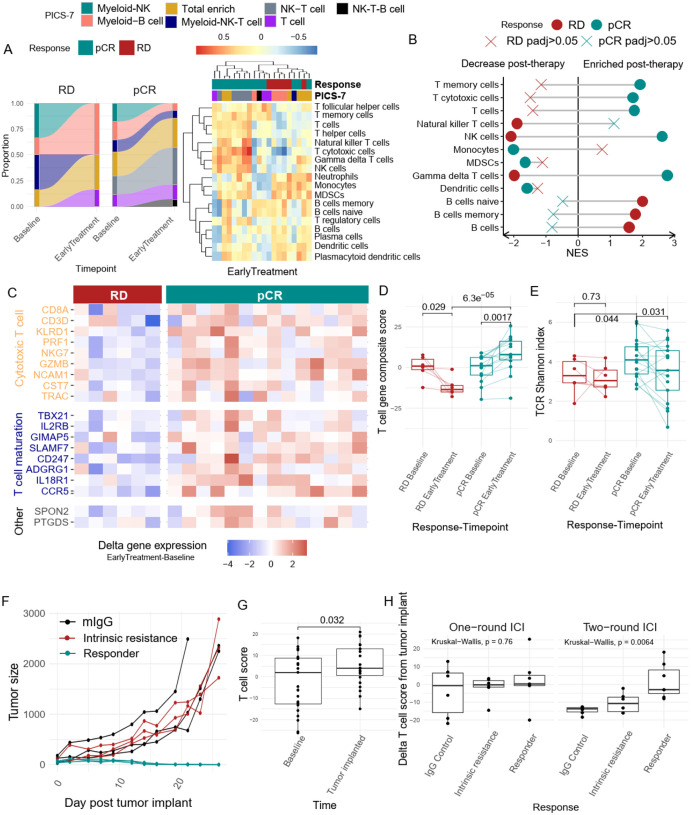
Peripheral T cell activation is associated with chemotherapy + pembrolizumab response in TNBC (**A**) TNBC Patients’ PICS-7 classification were compared before and after Chemo+Pembro treatment, the relative composition is measured separarely by response (left panel). Heatmap depicts the patients’ gene set enrichment score for the major immune cell subtype at EarlyTreatment,. Responders preferentially clustered together (right panel). (**B**) Differential gene expression analysis was performed between Baseline and EarlyTreatment Chemo+Pembro-treated TNBC patients followed by gene set enrichment analysis using immune cell signature. A positive net enrichment score indicates this specific immune cell expanded after treatment. The analysis was performed separately for pCR (responders, green) and RD(non-responders, red). (**C**) Heatmap depiction of the EarlyTreatment-Baseline delta expression matrix of the leading-edge genes contributed to T cell gene sets for Chemo+Pembro treated TNBC patients. The leading-edge genes are grouped by function, and the plot is separated by pCR (responders, green) and RD (non-responders, red). (**D**) 19 gene T cell composite score in Chemo+Pembro treated TNBC patients was compared between Baseline and EarlyTreatment, separated by response. Paired T test used to compare across timepoint, regular T test used for comparisons between pCR and RD patients. (**E**) Baseline TCR Shannon diversity index was compared between RD and pCR patients with T test. The TCR Shannon diversity index was also compared between Baseline and EarlyTreatment samples using paired T-test. **(F)** Tumor growth curve for EMT6 bearing mouse treated with anti-PDL1 therapy. The treatment start when tumor volume reaches 100mm^3^. Curves are colored by mouse’s response to therapy. (**G**) The 19 gene T cell composite score was calculated for each mouse’s peripheral blood bulk-RNAseq sample. The score was then compared between before and after tumor formation with paired-Wilcoxon test. (**H**) The 19 gene T cell composite score was compared after one round (left panel) and two rounds (right panel) of anti-PDL1 treatment across mouse that received control IgG treatment, mouse that did not respond to therapy, and mouse that responded to treatment. Kruskal Wallis test was applied.

In TNBC, NK-T cell and T cell clusters were exclusively observed in pCR patients at baseline, with no representation in RD patients [Figure 5A]. Following one cycle of Chemo + Pembro (T1), the proportion of pCR patients classified into the NK-T cell cluster notably increased, underscoring a strong association between NK-T cell enrichment and treatment response. In contrast, RD patients exhibited a rise in Myeloid–B cell clusters, with minimal presence of NK-T cells throughout [Figure 5A]. Further examination of detailed immune cell signatures revealed a widespread upregulation of lymphocyte signatures post-Chemo + Pembro, including genes characterizing T cells, cytotoxic T cells, and NK cells, in pCR patients [Figure 5B]. In contrast, B cell signatures, including genes associated with naïve and memory B cells, were enriched in RD patients, post-treatment. Additionally, monocyte, neutrophil, and MDSC-associated genes were uniformly downregulated in pCR patients, while no significant alterations were detected in RD patients [Figure 5B].

One key feature observed in TNBC Chemo + Pembro responders is the enrichment of T cell signatures. Therefore, we extracted the leading-edge genes of the GSEA enriched T cell subtypes to derive the potential immunophenotype predominating in these signatures. Among the 19 genes driving the T cell signature enrichment in pCR patients, we found multiple markers associated with T cell effector function or markers, such as GZMB, PRF1, NKG7 and KLRD1, and T cell maturation markers, such as TBX21 [Figure 5C]. We next calculated the difference in these gene signatures between early treatment and baseline and found that the majority of pCR cases experienced an upregulation of these 19 genes [Figure 5C]. To summarize this phenotype, we calculated a composite score of these genes and observed a uniform increase in this cytotoxic T cell score in pCR patients [Figure 5D]. The T cell composite score decreased in RD patients after treatment, which may be a sign of immune exhaustion and loss of effector function [Figure 5D].

We next assessed whether enrichment of cytotoxic markers was associated with clonal T cell expansion by analyzing dynamic TCR clonality using the Shannon diversity index. We found a significant decrease in the Shannon diversty index in post Chemo + Pembro pCR patients only, along with a higher level of TCR diversity in pCR patients compared to RD patients at baseline [Figure 5E]. Overall, our observation suggests that peripheral T cells in pCR patients carried diverse TCRs at baseline, experienced clonal expansion, and gained effector function after immunotherapy. In contrast, the T cells from RD patients were clonal at baseline and did not experience clonality changes after treatment.

Building on the observed T cell clonal expansion pattern post-Chemo + Pembro, we next compared the early treatment samples between pCR and RD patients to determine if there are notable differences in PBMC composition. As expected, we observed significant enrichment of multiple T cell subtypes and NK cells in pCR patients compared to RD patients **[Supplementary Fig. 5A]**. In contrast, B cells, dendritic cells, and monocytes were significantly enriched in RD patients **[Supplementary Fig. 5A]**. These differences in immune cell composition were sufficient to drive the clustering of pCR and RD patients at early treatment [Figure 5A]. Of particular interest, all but one pCR patient was assigned the T cell associated PICS-7 cluster while the Myeloid-B cell cluster was the dominant PICS-7 subtype in RD patients.

We sought to model increases in T cell composite scores observed in TNBC patients in mice and determine whether they were also associated with anti-tumor immune activity to ICIs. We utilized an immunotherapy responsive TNBC-like murine tumor model, EMT-6. Similar to humans, tumor-bearing mice demonstrate differential responses to anti-PD-L1 treatment, ranging from complete response to intrinsic resistance [Figure 5F]^25^. Longitudinal peripheral blood samples were collected at baseline (i.e. in healthy mice), after tumor implantation (i.e. tumor-bearing) and after two weekly doses of ICI treatment. Mice were followed for treatment outcome (i.e. complete response, or intrinsic resistance). RNAseq was then performed to examine peripheral immune signature changes to parallel our human studies. After tumor implantation, we observed a significant increase in the T cell composite score in the peripheral blood, supporting our observations of a systemic immune response based on the presence of the tumor [Figure 5G]. Interestingly, mice that responded to ICI treatment also demonstrated elevated levels of the peripheral T cell score compared to untreated mice, or those demonstrating ICI resistance [Figure 5H]. Thus, these data support the hypothesis that systemic immune features show a direct relationship to the anti-tumor immune response.

### Downregulation of leukocyte chemotaxis distinguishes Chemo + Pembro responders in HR + BC

**Figure 6:**
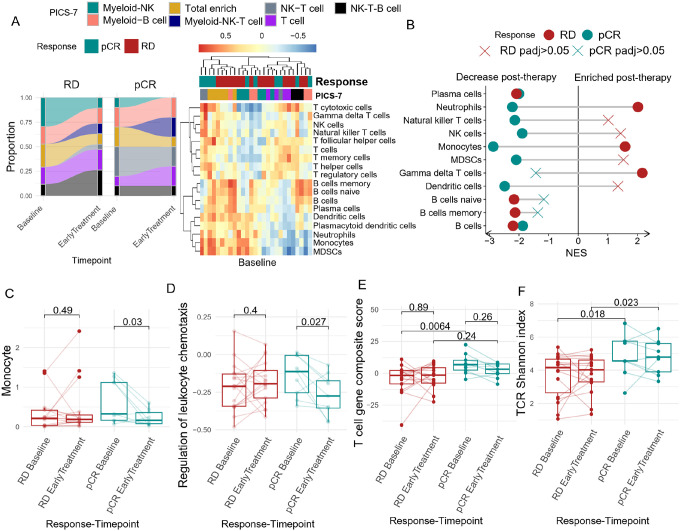
Baseline T cell signatures are associated with chemotherapy + pembrolizumab response in HR+BC (**A**) (**A**) HR+ Patients’ PICS-7 classification were compared before and after Chemo+Pembro treatment, the relative composition is measured separarely by response (left panel). Heatmap depicts the patients’ gene set enrichment score for the major immune cell subtype at EarlyTreatment,. Responders preferentially clustered together (right panel). (**B**) Differential gene expression analysis was performed between Baseline and EarlyTreatment Chemo+Pembro-treated HR+ BC patients followed by gene set enrichment analysis using immune cell signature. A positive net enrichment score indicates this specific immune cell expanded after treatment. The analysis was performed separately for pCR (responders, green) and RD(non-responders, red). **(C)** CIBERSORT deconvoluted monocyte (classical+non-classical) abundance was compared before and after Chemo+Pembro treatment in responders and non-responders **(D)** GOBP leukocyte chemotaxis score was calculated for each sample using GSVA. The enrichment score was compared before and after Chemo+Pembro treatment with paired T test. (**E**) 19 gene T cell composite score in Chemo+Pembro treated TNBC patients was compared between Baseline and EarlyTreatment, separated by response. Paired T test used to compare across timepoint, regular T test used for comparisons between pCR and RD patients. (**F**) Baseline TCR shannon diversity index was compared between RD and pCR patients with T test. The TCR Shannon diversity index was also compared between Baseline and EarlyTreatment samples using paired T-test.

We previously demonstrated differences between BC subtypes both at baseline and in response to therapy, suggesting that the underlying biology of the disease and its response to therapy (even at the systemic level) may result in divergent responses. In HR + BC, we noted a subtle stratification of responders and non-responders by immune cell enrichment at baseline. Notably, at baseline, the NK-T cell cluster (PICS-7 subtype) was exclusively detected in pCR patients [Figure 6A]. Differential enrichment analysis at baseline further revealed that T cell and NK cell-associated gene signatures were predominantly enriched in pCR patients, while B cell signatures were more prevalent in RD patients **[Supplementary Fig. 5B]**.

Detailed analysis of differentially enriched immune cell signatures post Chemo + Pembro revealed a significant decrease in myeloid cell marker expression in pCR patients and an increase in these markers in RD patients. Specifically, markers associated with monocytes, MDSCs, neutrophils, and DCs contributed to the change. Immune cell deconvolution corroborated these findings, showing a significant decrease in monocyte abundance (combining non-classical and classical monocytes) in pCR patients [Figure 4C], while no significant trend was detected in RD patients. We further explored other peripheral immunological features associated with the decrease in monocytes and found a concurrent decrease in the leukocyte chemotaxis score post-treatment in pCR patients [Figure 4D]. Given that the leukocyte chemotaxis score is significantly correlated with the tumoral chemokine score at baseline [**Supplementary Fig. 6**], this observation could suggest that Chemo + Pembro may modulate the tumor microenvironment in HR + BC by reducing monocyte recruitment via the peripheral compartment.

Although T cell immune signatures were not differentially enriched during therapy, we investigated whether the T cell composite score, previously defined for Chemo + Pembro treated TNBC patients, could reduce bias and help detect T cell changes in HR + BC during Chemo + Pembro treatment. Besides a higher baseline T cell composite score [Figure 6E] and higher baseline and early treatment TCR Shannon diversity index [Figure 6F] in pCR patients, we did not observe significant changes in T cell-associated metrics induced by Chemo + Pembro. Our findings indicate distinct immune activation mechanisms are induced by Chemo + Pembro in HR + BC compared to TNBC, highlighting the complexity and specificity of the immune response in different breast cancer subtypes.

### T cell activation status is exclusively associated with Chemo + Pembro response

Although various immune response signals and dynamics in immune cell composition were observed, only the higher baseline 19-gene T cell composite score (HR + BC specific), post-treatment enrichment of T cell composite score (TNBC specific), post therapy reduction in leukocyte chemotaxis (HR + BC specific), and baseline TCR clonality were exclusively associated with the Chemo + Pembro response [Figure 5D–E, Fig. 6E–F, **Supplementary Fig. 7A-D**]. To further test the predictive power of these markers, we generated a logistic regression model. This model incorporated the T cell composite score at Baseline (HR + BC), the reduction in leukocyte chemotaxis score (HR + BC), the increases in T cell composite score at EarlyTreatment (exclusive for TNBC), and the baseline TCR clonality, to distinguish chemotherapy and pembrolizumab responders from non-responders.

**Figure 7:**
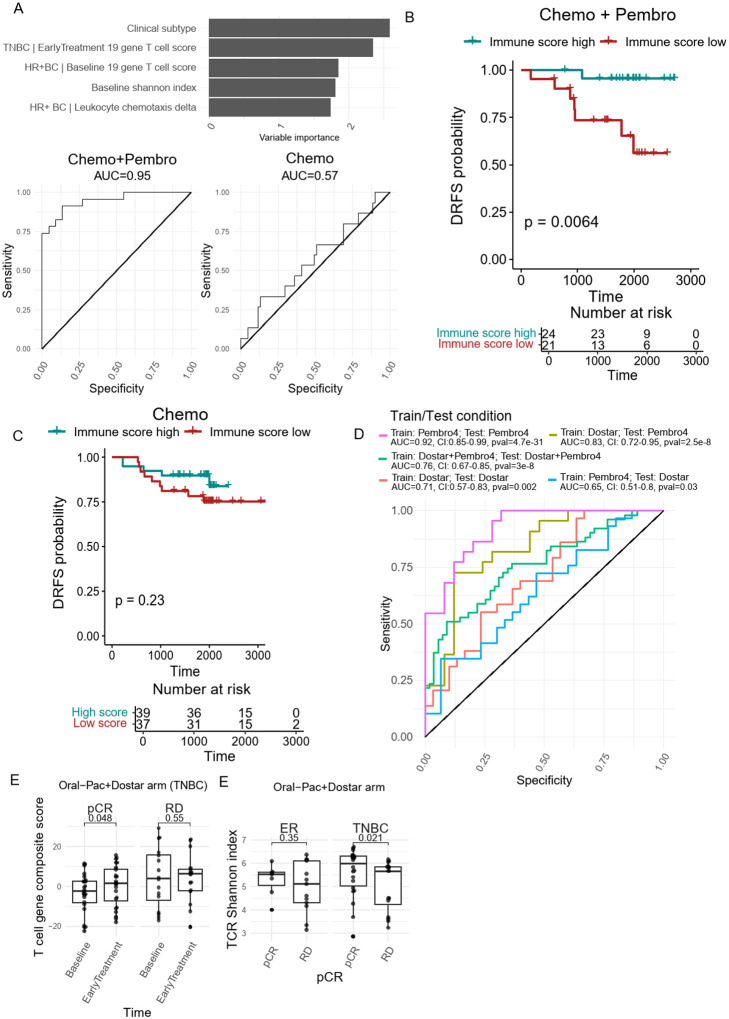
Peripheral immune biomarkers predicts chemo+Pembro response. **(A)** Logistic regression using breast cancer clinical subtype, 19 gene T cell signature, changes in leukocyte chemotaxis score and Baseline TCR Shannon diversity index was performed to predict patients’ response to Chemo+Pembro. We used the same training and testing data set, containing all patients treated with Chemo+Pembro. Kaplan–Meier analysis of DRFS for the **(B)**Chemo+Pembro and **(C)** Chemo arms of the I-SPY2 trial according to GLM prediction score based on peripheral immune signatures. Patients were classified as Immune high if the GLM obtained probability score >=0.45 for Chemo+Pembro and >=0.19 for Chemo. Cutoff based on optimal point on AUC. (**D**) ICI response prediction model validation was performed using various training and testing data combinations. The AUC for each condition is listed in the figure, along with with 95% confidence intervals and the pvalue of the AUC is significantly different from 0.5. (**E**) The TNBC patient’s 19 gene T cell composite score was compared before and after treatment. Paired T-test was performed separately for pCR and RD patients. (**F**) The baseline TCR Shannon diversity index was compared between pCR and RD patients. T-test was performed separately for HR+ BC and TNBC patients.

We performed training and testing on the same data set and found that the regression model trained on these peripheral immune matrices could effectively identify patients who responded to the treatment regimen [Figure 7A, **Supplementary Table 3**]. However, the model did not distinguish chemotherapy response. Model performance was further tested with 10-fold cross validation. The Kappa value was drastically higher in Chemo + Pembro than Chemo (0.64 vs 0), strengthening the association between those peripheral immune biomarker and Chemo + Pembro response. We next tested whether the peripheral immune signatures could identify long term benefit. We separated the patients as immune signature high versus low with an optimal cutoff from logistic regression ROC curve. When comparing the patient’s distance recurrence free survival (DRFS), patients with high immune score showed significantly improved DRFS when comparing to the low immune score cohort [Figure 7B]. This trend was not observed in the Chemo arm [Figure 7C].

It is acknowledged that training and testing on the same data set likely results in overfitting. We therefore performed validation on the Baseline and EarlyTreatment samples from 59 patients receiving oral paclitaxel + encequidar + dostarlimab. The model, when using data from Pembro4 (the original discovery set) as training set, maintained predictive capacity to dostarlimab combination therapy (AUC = 0.65, CI: 0.51–0.8, p = 0.03) [Figure 7D]. Thus, these findings demonstrate potential generalizability across multiple treatment arms.

When using data from the Dostar arm to train the coefficients of the model parameters and testing on the Pembro4 arm, the model achieved an AUC of 0.83 (CI: 0.72–0.95, p = 2.5e-8). A combined training on both cohorts, tested on the pooled set, achieves an intermediate AUC of 0.76 (CI: 0.67–0.85, p = 3e-8), suggesting robustness of the model when leveraging a more diverse training population. Overall, these results suggest that the Pembro4-trained model captures a biologically relevant signal that is, to some extent, transferable to patients receiving a distinct but mechanistically related immunotherapy regimen. Given the observed differences in predictive capacity when calculating coefficients on different training/test populations, we anticipate inclusion of additional data across more diverse populations of ICI-treated patients will help stabilize the model for the broadest and most reliable generalizability.

In addition, we observed an enrichment in T cell composite score in TNBC patients responding to oral paclitaxel + encequidar + dostarlimab, similar to the patients that responded the pembrolizumab [Figure 7E]. We also observed higher baseline TCR Shannon diversity index in TNBC responders [Figure 7F]. These observations indicate that baseline as well as on-treatment T cell response is associated with immunotherapy outcomes, and these immunological signatures could be generalized across different ICI regimen.

We also generate a TNBC-only model and a HR + BC-only model, each containing subtype-specific ICI response biomarker. We found that the model performance is similar between TNBC and the original model. Both models in general perform better than the HR + BC only model (higher numerical AUC). However, the ROC curves are not significantly different. This trend is independent of training and testing data set **[Supplement Fig. 8A]**. In addition, we tested whether the model performance would be different if only incorporating baseline features such as baseline T cell composite score or TCR clonality. We found that the simplified, baseline model’s ROC curve is not significantly different from the original **model Supplement Fig. 8B]**. This result suggests that, upon further validation, there is a potential to further simplify the prediction model.

## Discussion

The cancer immunity cycle^26^ suggests that antigens released from tumors during cell turnover and in response to therapies drain to the lymph node, where they are acquired by professional antigen presenting cells and presented to T lymphocytes for priming. Appropriately primed T cells are ‘licensed’ to leave the lymph node and seek out sources of inflammation, whereby they must pass through the systemic peripheral circulation as a conduit. Thus, there is biological impetus for sampling the peripheral immune system as a proxy for ongoing antitumor immunity.

**Figure 8:**
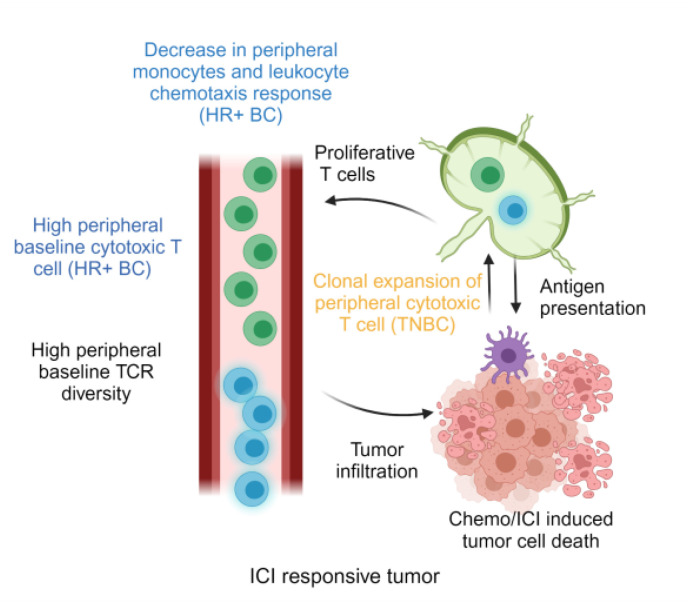
HR+BC and TNBC Chemo+Pembro responders experienced different systemic immune responses after one round of therapy.

In this study, we asked whether peripheral blood transcriptomic signatures of breast cancer patients before and after Chemo + Pembro could provide evidence of systemic anti-tumor immunity. Here, using > 500 RNA sequenced transcriptomes collected longitudinally from 160 patients, we demonstrated that the baseline systemic immune environment differed significantly by breast cancer phenotypes, including clinical subtypes, molecular subtypes, and tumoral immune microenvironment. In addition, those differences had further impact during Chemo + Pembro treatment and generated distinct biomarkers that are associated with therapeutic response [Figure 8].

TNBC (and more specifically, basal-like breast cancer) is the most immunogenic subtype potentially due to high tumor mutation burden^27^, elevated expression of major histocompatibility complex^25,28^, and associated increased tumor lymphocyte infiltration^21,29^. These immunogenic features create a highly inflamed baseline peripheral immune landscape and upon effective Chemo + Pembro treatment, induce systemic T cell clonal expansion and activation, similar to other immune-hot cancer types^15,30,31^.

The recent success of the KEYNOTE-756 trial has challenged the previous belief that immune-cold HR + BC would not respond to immunotherapy^4^. We found that Chemo + Pembro induced minimal changes in peripheral T cell activity, consistent with previous reports of similar observations in metastatic tumors^32^. Meanwhile, Chemo + Pembro responders experienced significant decrease in monocyte abundance and leukocyte chemotaxis score. The downregulation of myeloid signals and recruitment of myeloid cells may reflect a mechanism to remove immune suppression from tumor associated macrophages or myeloid derived suppressor cells. This unexpected therapeutic effect may be attributed to the reduction of the chemokines and cytokines responsible for myeloid cell recruitment in the tumor microenvironment^33–35^, which may be distinct from the classical mechanism of action observed in TNBC and other immune-hot tumor types..

We demonstrated that immunophenotype, particularly those longitudinal signatures captured in patients’ peripheral blood, can potentially predict response to immunotherapy. One major challenge in clinical biomarker studies using baseline samples is patient variability; factors like age, sex, and prior disease history can significantly bias biomarker development. Longitudinal sampling, such as the baseline-early treatment blood samples in our study, allows us to focus on therapy-induced changes rather than baseline patient characteristics. This approach is especially valuable in the context of immunotherapy, as the immune system evolves with the tumor during treatment. Therefore, future research should prioritize multi-timepoint time-series studies to identify potential signatures associated with treatment response.

Although the longitudinal peripheral blood data presented here is an unparalleled resource to study systemic immunity under immunotherapy, our study has several limitations. Further dissecting the cohort by clinical subtype, treatment arm, and treatment response results in small sample size and uneven group distributions. This limits the power of our analysis and the ability to reduce sample specific variants. In this study, we validated the ICI response prediction biomarkers in the oral-paclitaxel + dostarlimab arm in the I-SPY2 study. The model, although demonstrating potential generalizability across ICI treatments, experiencing variations in its performance potentially due to different treatment regimens, patient populations, or sample storage conditions and batch effects. Future studies should focus on integrating large dataset with high-power machine learning/deep learning models to integrate complex immune features and stratify patients more accurately. Altogether, these challenges limit our ability to draw definitive conclusions. Thus, our statistics are descriptive rather than inferential; and all individual predictors of response require further testing to assess their prediction characteristics within different treatment settings.

Even with immune cell deconvolution algorithm like CIBERSORT, a major limitation of this manuscript is the lack of high-resolution peripheral immune cell profiling. In our study, we used buffy coat blood samples, which are not optimal for live-cell-dependent methods like flow cytometry or single-cell RNAseq. This limitation is a key factor in our decision to focus on RNA-seq as the primary approach for immune profiling in this context. Expanding the scope of our work is a priority, and we are planning future prospective trials where we will collect cryo-preserved samples. This will allow for more robust multi-omic studies, including both transcriptomic and proteomic profiling, as well as incorporating other techniques such as flow cytometry.

The dataset presented in this study offers a unique opportunity to understand the connection between systemic and tumoral immune environment, especially under treatment perturbation. In addition, biomarkers discovered in this study, upon further validation, could serve as a novel ‘liquid biopsy’ to help predict therapeutic benefit, individualize therapeutic regimens, and aid in precision medicine in breast and potentially other cancers.

## Methods and materials

### Patient population

546 samples from 160 patients passed final quality control and were included in this study. Patients were treated with weekly paclitaxel for 12 weeks ± 4 rounds of pembrolizumab administered every 3 weeks. After initial treatment, additional neoadjuvant treatment of 4 cycles of doxorubicin (A) and cyclophosphamide (C) were administered. Patient selection criteria, dosage, and response has been previously reported in detail^2,18^. Longitudinal samples were collected at baseline, after 1 treatment cycle (3 weeks of paclitaxel ± pembrolizumab), after initial treatment completion, and after additional neoadjuvant AC prior to surgery. For the validation cohort, 139 samples from 77 patients passed final quality control. Among these samples, 58 patients has both Baseline and EarlyTreatment samples and were selected for subsequent validation study. Patients were treated with oral paclitaxel 205mg/m2 and encequidar 12.9 mg on days 1–3, carboplatin AUC 1.5 on day 1 weekly × 12, and dostarlimab 500 mg every 3 weeks × 4, followed by doxorubicin/ cyclophosphamide (AC) every 2–3 weeks × 4. Patient selection criteria, dosage, and response has been previously reported in detail^19^. I-SPY2 is conducted in accordance with the guidelines for Good Clinical Practice and the Declaration of Helsinki, with approval for the study protocol and associated amendments obtained from independent ethics committees at each site. Written, informed consent was obtained from each participant prior to screening and again prior to treatment. The I-SPY2 Data Safety Monitoring Board meets monthly to review patient safety.

### Buffy-coat peripheral blood collection

Within two hours of blood collection, tubes containing the whole blood were centrifuged at 1100–1300 g for 20 minutes at room temperature. The buffy coat layer (above the red blood cells) was removed and aliquoted into four 2 ml cryovials, with some red blood cells allowed in the buffy coat. Two buffy coat vials were frozen directly upright at −80°C.

### Bulk RNA sequencing and data preprocessing:

Total RNA was isolated from frozen buffy coat blood samples using the Maxwell^®^ 16 LEV simplyRNA Purification Kits, Blood (cat# AS1310) following the manufacturer’s instructions. Briefly, frozen buffy coat samples were thawed and went through red blood cell lysis. The remaining PBMC were lysed and homogenized to release RNA. Magnetic beads specific for capturing RNA were used to isolate RNA from cellular debris and other contaminants. Following washes to remove impurities, the purified RNA was eluted in water and quality control was performed with NanoDrop. Next, mRNA enrichment and cDNA library were prepared utilizing the stranded mRNA (polyA-selected) library preparation kit. Sequencing was performed at Paired-End 150 bp on the Illumina NovaSeq 6000 targeting an average of 50M reads per sample. Demultiplexed FASTQ files were next aligned using STAR with a genome index generated from human Hg38. FeatureCount was next applied to create gene count matrix. Subsequent MultiQC was performed to ensure sample homogeneity. In parallel, TCR sequence was extracted from raw FASTQ files with MixCR using default parameters based on the developers’ instructions (https://github.com/milaboratory/mixcr/). In brief, sequencing reads were aligned to reference V, D, J and C genes of T cell receptors. Then, the aligned reads were assembled to extract CDR3 gene regions. Finally, the clonotypes were exported in a human-readable/parsable tab-delimited text file format. Combat-Seq (R, sva package) was performed to remove potential batch effect from the discovery and validation dataset. Patient’s breast cancer subtype was used as a covariate during correction.

### Cell culture

EMT6 murine mammary carcinoma cells were cultured in DMEM/F12 (Thermo Fisher Scientific) supplemented with 10% FBS (Thermo Fisher Scientific).

### Orthotopic animal experiments

For murine models, EMT6 cell lines (5×104 cells) were injected in 100uL of PBS into the #4 mammary fat pad of 6-week-old BALB/c female mice. Tumor formation and growth was followed for up to 100 days. Tumors were measured 2 times weekly with calipers and volume was calculated in mm3 using the formula (width2 × length/2). Mice were humanely euthanized when their tumor burden reached 2000 mm3, at 100 days for survival studies, or at earlier timepoint for analysis. Blood from mice via the submandibular vein, before and after tumor inoculation and after every round of treatment.

### In vivo therapy regimens

Anti-PD-L1 (clone 6E11; Genentech) were administered at an initial dose of 200 ug intraperitoneally when individual mouse tumors reached 100 mm3 and subsequently dropped to 100ug weekly for up to 4-weeks of total administration time.

### Mouse peripheral blood bulk RNA sequencing

RNA was harvested from cells or PBMC using the Maxwell 16 automated workstation (Promega) using the LEV simplyRNA Tissue or Blood purification kit (Promega), respectively. RNA was analyzed for concentration by NanoDrop 2000 (ThermoFisher) Reads were trimmed to remove adapter sequences using Cutadapt v2.10) and aligned to the Gencode GRCm38.p6 genome using STAR (v2.7.8a). Gencode vM24 gene annotations were provided to STAR to improve the accuracy of mapping. Quality control on both raw reads and adaptor-trimmed reads was performed using FastQC (v0.11.9) (www.bioinformatics.babraham.ac.uk/projects/fastqc). featureCounts (v2.0.2)15 was used to count the number of mapped reads to each gene.

#### Quantification and statistical analysis

##### Differential gene expression analysis

Raw count generated by FeatureCount was imported to R. Genes that were expressed in less than 50% of the samples were excluded from the analysis. The filtered gene count matrix was next used to generate DESeq2 objects with corresponding metadata. Differentially expressed genes between clinical subtypes, molecular subtypes, and immunophenotypes were directly compared using DESeq2 function with design of ~*cl* ∈ *ical* ⊂ *type*, ~*mo* ≤ *c**a**r* ⊂ *type, ~Immμnophe* ¬*ype* + *cl* ∈ *ical* ⊂ *type*, respectively. The results from differential gene expression analysis were next feed into gene set enrichment analysis using fgsea based on Hallmark 50 gene pathways or Immune cell signatures derived from PangLaoDB database to capture enrichment of immune related pathways or immune cell subtypes.

Longitudinal comparisons were also performed using DESeq2 when monitoring differential treatment effect within each clinical subtype with or without further separation of the cohort by response. ~*Time* + *patientID* design function was used for all the longitudinal comparisons thus adjusting for patient specific variants. The results from differential gene expression analysis were then used in gene set enrichment analysis to identify alterations in immune related pathways and immune cell subtypes.

##### TCR clonality

After CDR3 region sequencing was extracted using MIXCR, for each sample, TCR clonality was calculated using Shannon diversity index (vegan, R package) based on the abundance of different CDR3 regions from TCR beta sequence. Samples with 0 Shannon diversity index or have less than 10 clones detected were removed from future analysis due to low sample quality.

###### Tumor correlation analysis:

8 continuous immune-related scores were previously calculated based on patient’s tumoral RNA profile^17^. All 8 immune-related scores, except mast cell score, are positively associated with therapy outcomes. However, the immune associated markers in different receptor subtypes seem to have different predictive biology: high dendritic, chemokine, and STAT1 cells/signals best predict response for TNBC, whereas high B-cell combined with low mast cell best predict pCR in HR + HER2−. We performed Pearson correlation analysis between the 8 continuous tumoral immune score with peripheral blood immune biomarker derived from Hallmark 50 gene pathway or PangLaoDB single cell immune cell reference. Additionally, a binary immune response variable, Immune+/−, was defined by unsupervised clustering of patient’s tumoral immune score^17^. To identify peripheral immunological differences between Immune+/− patients, differential gene expression analysis was performed using the aforementioned method.

Besides immune associated scores, breast cancer lineage and hormone receptor expression were also previously described in these tumors^17^. A basal/luminal lineage score (derived from BluePrint, Agendia), and ER/PGR expression calculated by the average of ESR1 and PGR expression in the tumor, were also available. We adopted those scores and further tested their association with peripheral immune cell composition with Pearson correlation.

##### Cibersort immune cell devoncolution

Reference gene matrix for major PBMC immune cell subtype is generated by a single cell RNA sequencing data collected from baseline HR + BC patients’ PBMC [paper under review]. 11 immune cell subtypes each containing 500 cells were provided as reference to CIBERSORTx using program default parameter. Immune cell types include 4 CD8 T cell subtypes (Naïve/CM, Early activation, GZMB + Cytotoxic and GZMK + cytotoxic), CD4 T cell, B cell, non-classical and classical monocytes, NK cell, and conventional and plasmacytoid DC. The reference matrix generated by CIBERSORTx is available upon request. Bulk RNAseq data was then deconvoluted using this self-build matrix to derive immune cell composition in absolute mode.

##### PICS-7 cluster

Raw gene expression matrix was transformed with VST function in DESeq2. The enrichment score of each immune cell gene sets from PangLaoDB database was calculated using GSVA based on the vst-transfromed gene matrix (R package). Hierarchical clustering was performed across all the samples collected at Baseline and EarlyTreatment based on their respective Euclidian distance calculated from based on the immune cell subtype gene set score. The binary trees were cut by request to generate 7 unique clusters based on their representation on heatmap. Each cluster was annotated by the immune cell supertype (NK cell, T cell, myeloid cell, and B cell) enriched in that cluster.

###### Prediction Model:

Logistic regression was performed using GLM function in R on Chemo + Pembro or Chemo treated patients with formula: “Response ~ Clinical subtype + EarlyTreatment 19 gene T cell score|TNBC + Baseline 19 gene T cell score|HR + BC + Baseline TCR Shannon + EarlyTreatment-Baseline Leukocyte chemotaxis score| HR + BC”. The discovery cohort, training cohort, and testing cohort are the same, taking the entire sample population.

##### Statistical analysis

For testing the differences between groups within a specific timepoint, two-sided or one-sided T test or wilcox-test was applied. For cross-timepoint comparisons, paired T test or wilcox-test was applied. GLM with binomial family was applied to calculate the association between immune cell composition and breast cancer subtype. All correlation analysis was done using Pearson correlation unless otherwise specified. Statistical analysis was performed in R (4.1.3)

## Supplementary Material

Supplementary Files

This is a list of supplementary files associated with this preprint. Click to download.

- SupplementTable1Patientmetadata.xlsx
- SupplementTable2PICS7.xlsx
- SupplementTable3model.xlsx
- SupplementFigure.pptx

## Data Availability

Raw sequencing data has been submitted to SRA [PRJNA798787]. Patient’s metadata is included in this paper as Supplementary Table 1. Each patient’s RID can be cross referenced with previously published I-SPY2 study^17^.

## Code Availability:

This paper does not report original code. Any additional information required to reanalyze the data reported in this work paper is available from the lead contact upon request.
